# Valorization of *Miscanthus *×* giganteus* hydrolysates into heme-enriched yeast biomass and extract using xylose-fermenting *Saccharomyces cerevisiae*

**DOI:** 10.1186/s40643-026-01133-1

**Published:** 2026-09-15

**Authors:** Suk-Chae Jung, Hyunjoon Oh, Sungjun Kim, Shuchi Singh, Vijay Singh, Soo Rin Kim, Ting Lu, Hyun Gi Koh, Yong-Su Jin

**Affiliations:** 1 Department of Chemical and Biomolecular Engineering, University of Illinois, Urbana-Champaign, Urbana, IL 61801 USA; 2 Department of Food Science and Human Nutrition, University of Illinois, Urbana-Champaign, Urbana, IL 61801 USA; 3 DOE Center for Advanced Bioenergy and Bioproducts Innovation, University of Illinois, Urbana-Champaign, Urbana, IL 61801 USA; 4 Department of Agricultural and Biological Engineering, University of Illinois, Urbana-Champaign, Urbana, IL USA; 5 School of Food Science and Biotechnology, Kyungpook National University, Daegu, 41566 Republic of Korea; 6 Carl R. Woese Institute for Genomic Biology, University of Illinois, Urbana- Champaign, Urbana, IL 61801 USA; 7 Department of Bioengineering, University of Illinois, Urbana-Champaign, Urbana, IL 61801 USA; 8 Department of Biological and Chemical Engineering, Hongik University, Sejong, 30016 Republic of Korea; 9 Centre for Precision Fermentation and Sustainability (PreFerS), Illinois Advanced Research Center at Singapore (Illinois ARCS), Singapore, Singapore

**Keywords:** Lignocellulosic hydrolysate, Heme-enriched yeast extract, Xylose utilization, Fed-batch fermentation, *Saccharomyces cerevisiae*

## Abstract

Heme-enriched microbial products represent value-added bioproducts that can be produced through sustainable fermentation processes. Current microbial heme production platforms rely mainly on refined sugars and downstream purification, limiting cost-effectiveness. Here, we developed a lignocellulosic hydrolysate-based bioprocess for producing heme-enriched yeast extract using *Saccharomyces cerevisiae*. The xylose-fermenting *S. cerevisiae* SR8 accumulated approximately twofold higher specific intracellular heme on xylose than on glucose, reaching 0.25 ± 0.02 mg/g dry cell weight (DCW) compared with 0.13 ± 0.01 mg/g DCW, consistent with reduced Crabtree repression and enhanced respiratory metabolism. During fed-batch cultivation with *Miscanthus* × *giganteus* hydrolysates, residual glucose control suppressed ethanol overflow and promoted biomass formation, resulting in 17.5 ± 0.4 g/L DCW, a specific heme content of 0.20 ± 0.01 mg/g DCW, and a heme titer of 3.4 ± 0.1 mg/L. This performance exceeded that of a mock hydrolysate with identical sugar composition. Autolysis of the resulting biomass produced a lyophilized yeast extract containing 36 ± 2 µg heme/g, exceeding the heme content of the commercial yeast extract tested and supported comparable microbial growth. These results indicate that downstream heme recovery during yeast extract production remains a key bottleneck for improving the overall process efficiency and product heme density. Among the downstream extraction methods tested, high-pressure homogenization most effectively enhanced heme release. Overall, these results demonstrate lignocellulosic hydrolysate valorization into heme-enriched yeast extract and identify downstream recovery as a key target for process optimization.

**Graphical abstract:**

**Figure:**
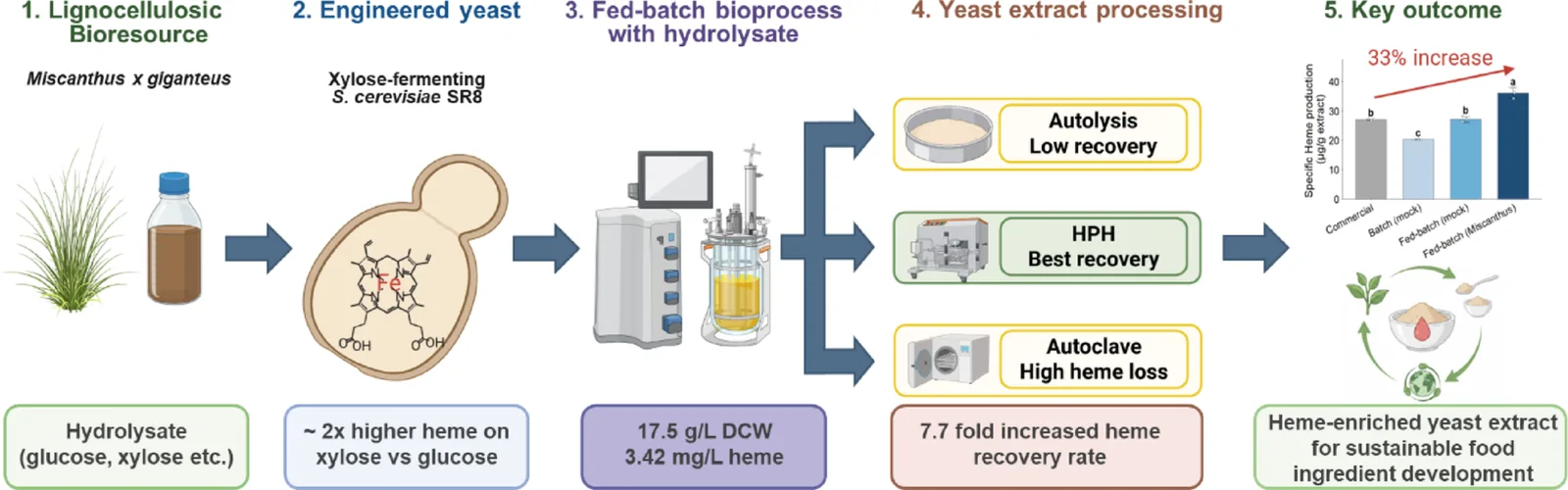

**Supplementary Information:**

The online version contains supplementary material available at https://doi.org/10.1186/s40643-026-01133-1.

## Introduction

Heme is gaining attention as a value-added microbial product because of its biological functions, pigmentation properties, and involvement in flavor-related reactions (Andreani et al. 2023). Structurally, heme consists of protoporphyrin IX and ferrous iron (Reedy and Gibney 2004). In eukaryotic cells, including animal muscle tissue, most iron is present in a heme-bound form, rather than as free iron (Dutt et al. 2022). Beyond its physiological roles in oxygen transport and protection against oxidative damage (Shimizu et al. 2019), heme is also relevant to pigmentation and flavor-related reactions in biological and food-associated systems (Ahmad et al. 2022). During heating, heme proteins can promote the formation of Maillard reaction-derived volatiles, whereas free iron may favor oxidized lipid notes (Li et al. 2024). These properties have motivated interest in scalable and sustainable microbial heme production (Rice et al. 2025).

Several microbial host strains have been engineered to overproduce heme using different strategies. The highest heme titer has been reported in *Escherichia coli*, reaching up to 1.03 g/L (Zhao et al. 2018; Choi et al. 2022). Secretion of intracellular heme could simplify recovery. In *Corynebacterium glutamicum*, 0.243 g/L, which is about 80% of the total produced heme, was secreted by overexpression of a putative heme transporter (Ko et al. 2021). Another strategy involves binding heme into a hemoprotein and secretion of the heme-binding protein. In *Komagataella phaffii*, 93% of the heme is bound to leghemoglobin, reaching a titer up to 3.5 g/L of leghemoglobin (Shao et al. 2022). However, these production platforms are typically designed for fermentation on refined sugars followed by downstream purification, which can increase process complexity and cost (Lee et al. 2024).

In contrast, *S. cerevisiae* offers advantages as a well-established and widely used microbial production host. *S. cerevisiae* biomass and yeast extract are already used as nutrient-rich yeast-derived products in fermentation, biotechnology, and food-associated applications (Tomé 2021; Wang et al. 2022; Kale et al. 2022). Accordingly, increasing intracellular heme content in *S. cerevisiae* provides a route to heme-enriched yeast extracts without requiring heme purification. Previous studies have demonstrated metabolic engineering strategies to enhance heme biosynthesis in *S. cerevisiae* (Ishchuk et al. 2022; Lee et al. 2024, 2025; Won et al. 2025). However, hydrolysate-based cultivation strategies for heme production using *S. cerevisiae* and downstream processing methods that retain intracellular heme in yeast-derived products remain underexplored.

Feedstock cost is one of the dominant cost contributors in precision fermentation (Zoghlami and Paës 2019). Therefore, utilizing abundant, low-cost alternatives to refined sugars such as lignocellulosic hydrolysates derived from non-food biomass is promising. However, efficient and rapid utilization of xylose, a major sugar component of lignocellulose, is essential for sustainable and economical conversion into value-added products (Koh et al. 2024). Xylose metabolism in *S. cerevisiae* can be enabled through heterologous expression of xylose reductase (XR), xylitol dehydrogenase (XDH), and xylulokinase (XK), which channel xylose into central carbon metabolism (Kim et al. 2013; Kwak and Jin 2017). Unlike glucose, which induces the Crabtree effect and promotes fermentative growth, xylose utilization favors respiratory metabolism and mitochondrial activity (Jin et al. 2004; Ha et al. 2013). Because several steps of heme biosynthesis occur in mitochondria and are coupled to respiration (Zhang et al. 2017), xylose metabolism provides a favorable physiological state for enhanced intracellular heme accumulation.

Lignocellulosic hydrolysates typically contain mixed sugars, including glucose and xylose (Banerjee et al. 2024). When glucose and xylose are present simultaneously, this can induce ethanol overflow metabolism in *S. cerevisiae* and potentially limit respiratory heme biosynthesis (Ha et al. 2013). Fed-batch fermentation is commonly used to control sugar availability, reduce ethanol formation, and promote respiratory metabolism in mixed-sugar systems (Patkar and Seo 1992; Halka et al. 2018). Applying fed-batch strategies to the fermentation of lignocellulosic hydrolysates may therefore enhance heme accumulation in xylose-utilizing yeast.

Beyond strain-level heme production, heme retention during downstream yeast extract preparation must also be evaluated, as this step determines how much of the intracellular heme is ultimately recovered in the final ingredient. Two factors must be considered. The harvested biomass must be sufficiently disrupted to release heme, as incomplete cell opening leaves heme partitioned in the discarded insoluble debris. Disruption conditions must not degrade the released heme. Commercial yeast extract is typically produced by autolysis. However, the commercial yeast extract preparation is not optimized for heme recovery or retention. Thus, further optimization for heme-enriched yeast extract preparation is warranted.

To address this gap, we aimed to establish an integrated process that converts non-food lignocellulosic hydrolysates directly into a heme-enriched yeast extract, using the food-compatible xylose-fermenting *S. cerevisiae* SR8 (Kim et al. 2013). SR8 is engineered for xylose assimilation but carries no additional heme-overproduction pathways, so any increase in intracellular heme reflects native biosynthesis under the tested conditions rather than pathway engineering. We first demonstrated that xylose drives higher intracellular heme accumulation than glucose, owing to the respiratory metabolic state required for mitochondrial heme biosynthesis. Building on this, a fed-batch bioreactor strategy was used to limit glucose-driven overflow metabolism during fermentation of *Miscanthus × giganteus* hydrolysates, which contain both glucose and xylose. Yeast extract derived from the hydrolysate-grown biomass contained more heme than the commercial yeast extract tested, while supporting microbial growth comparably as a nutrient source. This growth-supporting assay was used only to evaluate the nutrient functionality of the yeast extract and was not intended to demonstrate performance in a food matrix. Critically, we found that a major bottleneck in producing heme-enriched yeast extract lies in heme recovery during the yeast extract production process. Although further optimization is necessary, high-pressure homogenization released substantially more heme into the soluble fraction than autolysis. Together, these results demonstrate the valorization of lignocellulosic hydrolysates through the production of heme-enriched yeast extract and identify downstream heme recovery as a key target for improving hydrolysate-based bioprocesses.

## Materials and methods

### Strains and media

For the fermentation experiments, the *S. cerevisiae* SR8 strain was employed (Kim et al. 2013). For flask fermentations, YP medium (10 g/L yeast extract, 20 g/L peptone) supplemented with different sugars (40 g/L glucose, 40 g/L xylose, and a mixed sugar of 40 g/L glucose and 40 g/L of xylose) was used. For bioreactor experiments, the yeast-defined medium described by a previous report (van Hoek et al. 2000) was used for batch and fed-batch fermentation. The batch culture medium contains a hydrolysate-mimicking sugar mixture (25 g/L glucose, 21 g/L xylose, and 2 g/L acetic acid), 15 g/L (NH_4_)_2_SO_4_, 8 g/L KH_2_PO_4_, 0.72 g/L ZnSO_4_·7H_2_O, 6.15 g/L MgSO_4_·7H_2_O, 12 mL of vitamin solution per L, and 10 mL of trace metal solution per L. Trace metal solution contained 15 g/L EDTA, 10.2 g/L ZnSO_4_·7H_2_O, 0.50 g/L MnCl_2_·4H_2_O, 0.5 g/L anhydrous CuSO_4_, 0.86 g/L CoCl_2_·6H_2_O, 0.56 g/L Na_2_MoO_4_·2H_2_O, 3.84 g/L CaCl_2_·2H_2_O and 5.12 g/L FeSO_4_·7H_2_O. Vitamin solution contained 0.05 g/L biotin, 1 g/L calcium pantothenate, 1 g/L nicotinic acid, 25 g/L myoinositol, 1 g/L thiamine HCl, 1 g/L pyridoxal HCl, and 0.2 g/L 4-aminobenzoic acid. The fed-batch culture medium contains 15 g/L (NH_4_)_2_SO_4_, 8 g/L KH_2_PO_4_, 0.72 g/L ZnSO_4_·7H_2_O, 6.15 g/L MgSO_4_·7H_2_O, 12 mL of vitamin solution per L, and 10 mL of trace metal solution per L. The feeding solution contained a hydrolysate-mimicking sugar mixture (76 g/L glucose, 64 g/L xylose, and 7 g/L acetic acid) or *Miscanthus × giganteus* hydrolysate containing 76 g/L glucose, 64 g/L xylose, and 7 g/L acetic acid, 9 g/L KH_2_PO_4_, 3.5 g/L K_2_SO_4_, 0.28 g/L Na_2_SO_4_, and 5.12 g/L MgSO_4_·7H_2_O.

### Preparation of *Miscanthus *×* giganteus* hydrolysate

The *Miscanthus × giganteus* hydrolysate was prepared as previously reported (Banerjee et al. 2022, 2024; Cheng et al. 2023). Briefly, *Miscanthus × giganteus* was grown in experimental plots at the University of Illinois Energy Farm (Urbana, IL, USA) and harvested in December 2022. After harvest, the biomass was shredded and dried in a convection oven at 49 °C to a moisture content below 5% (w/w), followed by sequential hydrothermal–mechanical refining (HMR) pretreatment. The HMR process comprised chemical-free hydrothermal pretreatment and subsequent disc milling. Pilot‑scale hydrothermal pretreatment was carried out in a continuous reactor (SüPR-2G Hydrolyzer system, AdvanceBio Systems LLC, USA) after adjusting biomass moisture to 50% (dry basis), as described previously (Cheng et al. 2019, 2023). The pretreatment was conducted at 170 °C for 10 min. The resulting solids were dried at 40 °C to approximately 10% (w/w) moisture content and disc‑milled using a pilot‑scale burr mill (Meadows Mills, USA) configured to produce a 3 mm particle size (Banerjee et al. 2024). The pretreated biomass was stored in sealed containers at 4 °C until use. Enzymatic hydrolysis was conducted at 40% (w/v) solids loading using a fed‑batch strategy to mitigate substrate inhibition, with cellulase added at 50 FPU/g dry substrate (NS22257, Novozymes, USA) and hemicellulase at 3.75 mg protein/g dry substrate (NS22244, Novozymes, USA). Hydrolysis reactions were performed in 500 mL Erlenmeyer flasks in an incubator shaker at 60 °C and 180 rpm for 96 h.

### Flask fermentation

Batch shake-flask fermentations were performed in 250 mL flasks containing 50 mL of YP medium supplemented with either 40 g/L glucose, 40 g/L xylose, or a mixed-sugar formulation (40 g/L glucose and 40 g/L xylose). Cultures were incubated at 30 °C with agitation at 250 rpm. Fed-batch shake-flask fermentations were carried out under the same temperature and agitation conditions using mixed sugars. The culture was initiated with 45 mL of YP and 1 mL of a feeding solution containing glucose and xylose (40 g/L each). Once ethanol was depleted, a 1 mL aliquot of the feeding solution was added, and this feeding step was repeated.

### Bioreactor fermentation

Batch and fed-batch fermentations were conducted in a 2 L bioreactor (Bioflo 115, New Brunswick, USA) under tightly controlled conditions. The process was operated at 30 °C with an agitation rate of 500 rpm and an aeration rate of 1 volume of air per volume of medium per minute (1 VVM). The pH was maintained at 5.5 using a pH-stat control system (deadband: 0.2) that automatically added 8% ammonia solution as needed. For batch fermentation, the full feed-equivalent sugar mixture was supplied at the beginning of cultivation. For fed-batch fermentation, 100 mL of feeding solution was added at 0, 6, 12, 18, and 24 h, for a total feed volume of 500 mL.

### Quantification of heme

Heme determination was performed using a modified spectrophotometric assay as previously described (Liu et al. 2014; Martínez et al. 2015; Ishchuk et al. 2022). Protoporphyrin IX (PPIX) was used as a standard material. To measure the concentrations of heme and PPIX in cells, 1 mL culture with cells corresponding to 10 OD_600_ units was harvested in amber tubes by centrifugation at 16,200 × g for 10 min (accuSpin Micro 17, Fisher Scientific, USA). Then, 500 µL of supernatant was transferred to new amber tubes. The collected cells were washed twice with double-distilled water. 500 µL of 20 mM oxalic acid was added to the collected cells and supernatants, and the mixture was vortexed. Solutions were incubated at 4 °C overnight. After incubation, 500 µL of preheated 2 M oxalic acid was added. Half of the resulting mixture was transferred to a new amber tube and heated at 98 °C for 30 min, while the other half was incubated at room temperature. The mixtures were then centrifuged at 16,200 × g for 10 min. 200 µL of supernatant from non-heated and heated samples were transferred into a transparent flat-bottom 96-well plate. Absorbance at 404 nm was measured using a microplate reader (BioTek Synergy 2, BioTek Instruments, USA) to quantify PPIX and heme (Sinclair et al. 1999). Heating the protoporphyrin rings in an oxalic acid solution removes the iron ions from the heme. Non-heated samples were used to quantify PPIX, whereas heated samples reflected the combined signal from PPIX and heme. Accordingly, heme content was calculated as the difference between the heated and non-heated measurements. To quantify heme in the cell extracts, 100 mg of each extract was transferred into 1.5 mL amber microcentrifuge tubes. Heme was then extracted using the oxalic acid method as described above. Commercial yeast extract was purchased from Fisher Scientific (USA).

### Production of yeast extract by autolysis

Yeast cells from the high-cell-density fed-batch fermentation of *Miscanthus × giganteus* hydrolysate were harvested by centrifugation at 4,700 × g (RCFmax) for 15 min at 4 °C using a SORVALL^®^ RC-3 C Plus centrifuge (Thermo Scientific, USA). The resulting cell pellets were subsequently washed three times with double-distilled water. The washed cell pellets were reconstituted in double-distilled water and subjected to autolytic digestion in a bioreactor (Bioflo 115, New Brunswick, USA) maintained at 50 °C, pH 6.0, and 300 rpm for 20 h to break down intracellular constituents (Tanguler and Erten 2008). Following autolysis, the lysate was separated by centrifugation at 4,700 × g (RCFmax) for 15 min at 4 °C. The resulting supernatant was clarified through sterile filtration with a 0.22 μm membrane filter. The filtered supernatant was subsequently lyophilized using a freeze-dryer (Labconco, USA) to obtain a stable powdered cell extract.

### Compositional analysis of yeast extract

The composition of yeast extract was examined by measuring soluble protein, amino nitrogen, total carbohydrate, and total nucleic acid contents. Lyophilized yeast extract was dissolved in double-distilled water (DDW) at 5 mg/mL, vortexed for 1–2 min, and centrifuged at 21,100 × g for 5 min at 4 °C (Centrifuge 5425R, Eppendorf, Germany). The resulting supernatant was used as a working stock. Samples were diluted to 1 mg/mL for soluble protein and amino nitrogen analyses and to 0.5 mg/mL for total carbohydrate and nucleic acid analyses. All assays were performed in technical triplicate using three biological replicates, and results were expressed on a dry-weight basis.

Soluble protein content was determined by the bicinchoninic acid (BCA) assay (Smith et al. 1985; Tao et al. 2023). Briefly, 25 µL of sample (1 mg/mL) or bovine serum albumin (BSA) standard (0–2000 µg/mL) was mixed with 200 µL of working reagent (Reagent A : Reagent B = 50 : 1, prepared within 1 h of use; Pierce BCA Protein Assay Kit, Thermo Fisher Scientific, USA) in a 96-well plate. The plate was incubated at 37 °C for 30 min, cooled to room temperature, and absorbance was measured at 562 nm using a microplate reader (BioTek Synergy 2, BioTek Instruments). Soluble protein content was expressed as mg protein per g of yeast extract.

Amino nitrogen content, expressed as glycine equivalents, was determined by the ninhydrin colorimetric assay (Lie 1973; Friedman 2004). A 100 µL aliquot of sample (1 mg/mL) or glycine standard (0–800 µg/mL) was mixed with 100 µL of freshly prepared ninhydrin reagent (2% w/v ninhydrin in ethylene glycol monomethyl ether, combined 1 : 1 with 4 M sodium acetate buffer at pH 5.5 immediately before use) in heat-resistant glass tubes. Tubes were heated at 100 °C for 15 min, immediately quenched in an ice-water bath for 5 min, and diluted with 500 µL of 50% (v/v) ethanol. A 200 µL aliquot was transferred to a 96-well plate and absorbance was measured at 570 nm. Amino nitrogen content was expressed as mg glycine equivalent per g of yeast extract.

Total carbohydrate content was determined by the phenol–sulfuric acid method (Masuko et al. 2005). A 200 µL aliquot of sample (0.5 mg/mL) or D-glucose standard (0–200 µg/mL) was placed in glass tubes and mixed with 200 µL of 5% (w/v) phenol solution. Subsequently, 1.0 mL of concentrated sulfuric acid was added rapidly and the mixture was allowed to stand at room temperature for 30 min for color development. A 200 µL aliquot was transferred to a 96-well plate and absorbance was measured at 490 nm. Total carbohydrate content was expressed as mg glucose equivalent per g of yeast extract.

Total nucleic acid content was estimated spectrophotometrically. Yeast extract samples diluted to 0.5 mg/mL were measured for absorbance at 260 nm and 280 nm using a NanoDrop spectrophotometer (Thermo Fisher Scientific, USA). Total nucleic acid concentration was calculated using the standard conversion factor in which an absorbance of 1.0 at 260 nm corresponds to 40 µg/mL of mixed nucleic acid.

### Statistical analysis

All cultivations were performed in triplicate, and data are presented as mean ± standard deviation. Pairwise comparisons (e.g., glucose vs. xylose, batch vs. fed-batch, mock vs. *Miscanthus × giganteus* hydrolysates) were analyzed using two-tailed Student’s *t*-test. Multi-group comparisons (e.g., glucose vs. xylose vs. mixed sugars; heme content across the four yeast extract preparations) were analyzed by one-way ANOVA followed by Tukey’s HSD post-hoc test. Statistical significance was set at *p* < 0.05 and is denoted in figures by asterisks (* *p* < 0.05; ** *p* < 0.01; *** *p* < 0.001) or by different lowercase letters above bars indicating significantly different groups. Statistical analyses were performed in R (version 4.5.1) using the stats and multcomp packages.

### Comparison of cell disruption methods for yeast extract production

To compare the effects of different cell disruption methods on yeast extract yield and heme recovery, yeast biomass obtained from flask cultivation with YPD (10 g/L yeast extract, 20 g/L peptone, and 20 g/L glucose) was subjected to three extraction methods: autolysis, autoclave-assisted extraction, and high-pressure homogenization (HPH). For all methods, harvested cells were washed three times with double-distilled water (DDW) and resuspended in DDW to the same biomass concentration before extraction.

For autolysis, the cell suspension was incubated in a shaking water bath incubator (C76 WATER BATH SHAKER, New Brunswick Scientific, USA) at 50 °C and pH 6.0 with agitation at 100 rpm for 20 h. After autolysis, the lysate was centrifuged at 4,700 × g (RCFmax) for 15 min at 4 °C to separate the soluble fraction from the insoluble cell debris. The resulting supernatant was collected as the autolysis-derived yeast extract fraction.

For autoclave-assisted extraction, the washed cell suspension was thermally disrupted by autoclaving at 121 °C for 15 min. After cooling to room temperature, the treated suspension was centrifuged at 4,700 × g (RCFmax) for 15 min at 4 °C, and the supernatant was collected as the autoclave-derived yeast extract fraction.

For HPH, the washed cell suspension was disrupted using a high-pressure homogenizer (EmulsiFlex-C3, AVESTIN, Canada) at 20,000 psi for 10 passes. This approach was selected based on previous applications of high-pressure homogenization for mechanical disruption of *S. cerevisiae* cells and yeast extract production (Spiden et al. 2013; Dimopoulos et al. 2020). To minimize heat accumulation during processing, the homogenized sample exiting the homogenizer was collected in a stainless-steel beaker placed on ice. The disrupted suspension was then centrifuged at 4,700 × g (RCFmax) for 15 min at 4 °C, and the supernatant was collected as the HPH-derived yeast extract fraction.

For each extraction method, the soluble supernatant was filtered through a 0.22 μm membrane filter and lyophilized using a freeze-dryer to obtain powdered yeast extract. Yeast extract yield was calculated as the dry weight of the lyophilized extract divided by the initial dry cell weight used for extraction. Heme contents in the initial biomass, soluble extract fraction, and insoluble debris fraction were quantified using the oxalic acid-based spectrophotometric assay described in Sect.  2.5. Apparent heme recovery was calculated as the total heme amount recovered in the lyophilized yeast extract divided by the total heme amount present in the initial biomass. Heme distribution between the soluble extract and insoluble debris fractions was used to evaluate heme partitioning during yeast extract production.

## Results and discussion

### Xylose enhances heme biosynthesis and cell growth relative to glucose

**Fig. 1:**
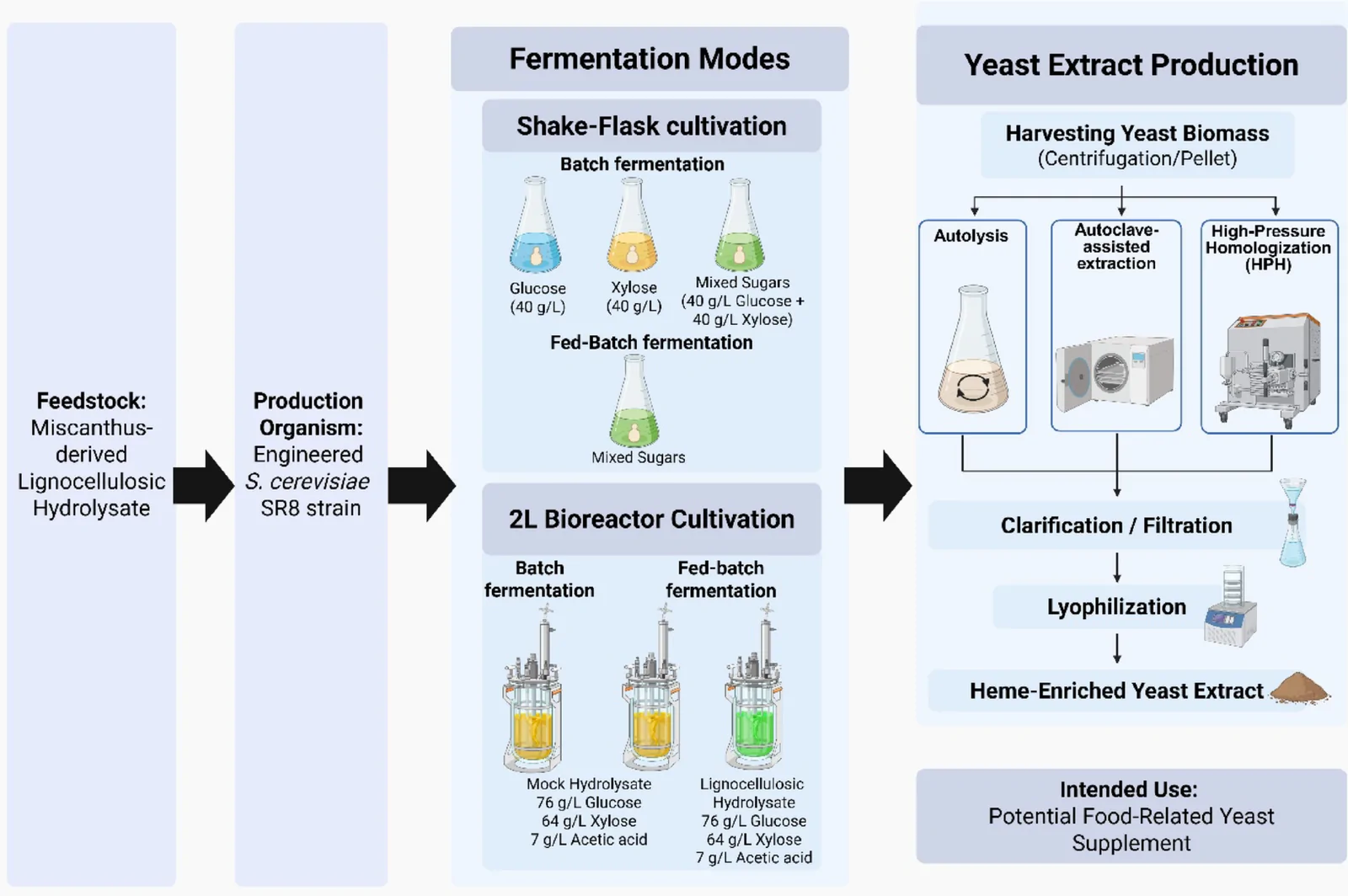
Overview of hydrolysate-based heme-enriched yeast extract production. Miscanthus-derived lignocellulosic hydrolysate was used as the feedstock for the cultivation of the engineered *S. cerevisiae* SR8 strain. Fermentation performance was evaluated across cultivation modes, including shake-flask batch fermentations on glucose (40 g/L), xylose (40 g/L), or mixed sugars (40 g/L glucose + 40 g/L xylose), as well as shake-flask fed-batch fermentation on mixed sugars. Scale-up was performed in a 2-L bioreactor using a mock hydrolysate formulation under batch and fed-batch operation, and lignocellulosic hydrolysate under fed-batch operation. Following fermentation, yeast biomass was harvested and processed into yeast extract by autolysis (50 °C, pH 6.0), clarification (centrifugation and filtration), and lyophilization to obtain a heme-enriched yeast extract as a potential food-related supplement

When Crabtree-positive *S. cerevisiae* consumes glucose, excess glucose induces respiro-fermentative metabolism even under aerobic conditions, leading to ethanol production. In contrast, xylose utilization, particularly in engineered *S. cerevisiae*, is associated with weaker repression of respiratory metabolism and reduced ethanol formation, thereby increasing biomass yield (Jin et al. 2004; Scalcinati et al. 2012). Because the engineered *S. cerevisiae* SR8 strain can efficiently consume both glucose and xylose (Kim et al. 2013), we compared biomass formation and heme accumulation under glucose- and xylose-based fermentations (Fig. 1).

**Fig. 2:**
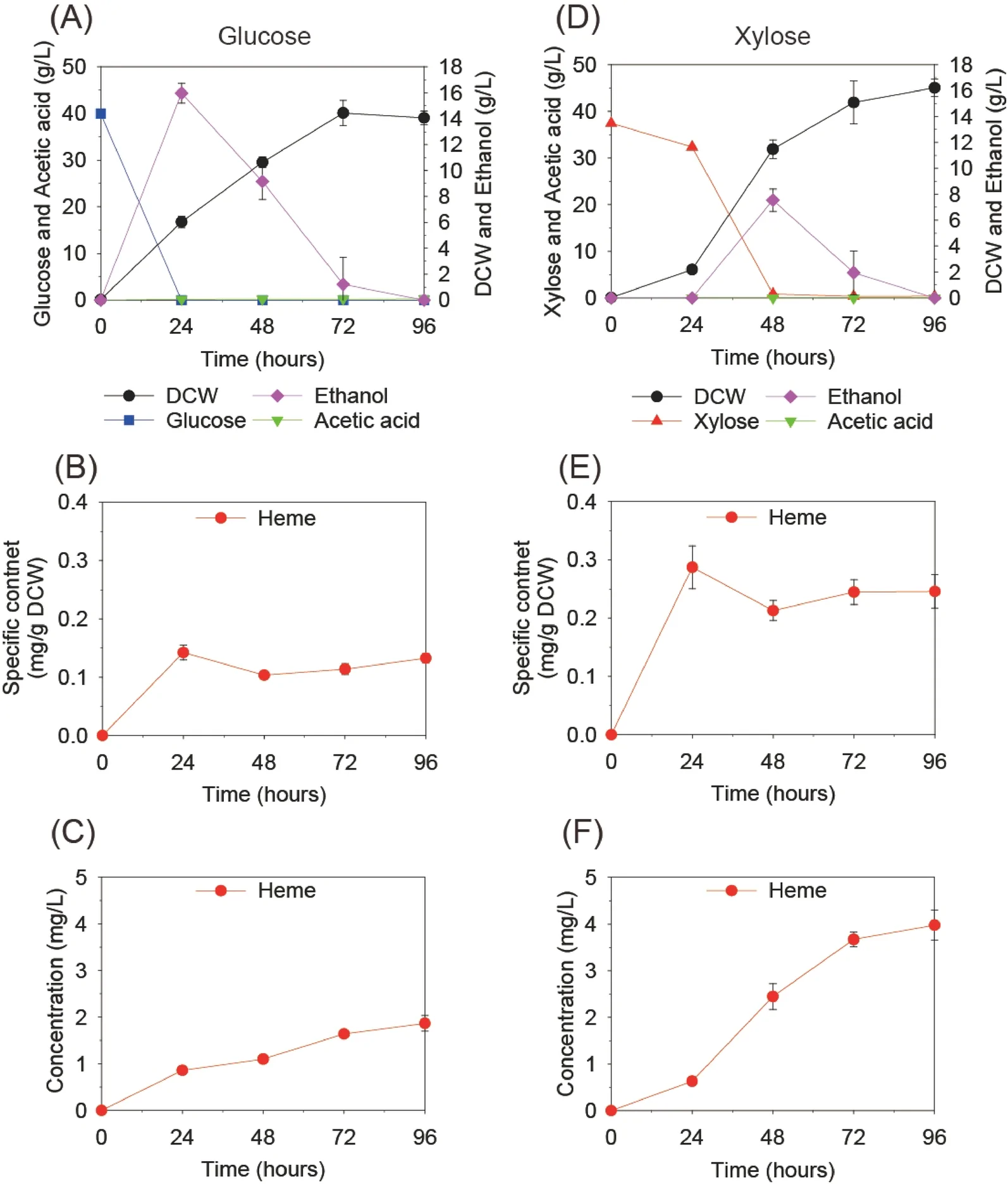
Xylose enhances biomass formation and heme production compared with glucose in shake-flask fermentation. Shake-flask batch fermentations of *S. cerevisiae* SR8 were conducted with either glucose (**A**–**C**) or xylose (**D**–**F**) as the sole carbon source. Time-course fermentation profiles on glucose (**A**) and xylose (**D**). Specific heme content on glucose (**B**) and xylose (**E**), volumetric heme concentration on glucose (**C**) and xylose (**F**) during fermentation of *S. cerevisiae* SR8. Experiments were performed in triplicate, and error bars represent standard deviations from each cultivation

SR8 accumulated greater biomass when grown on xylose than on glucose (Fig. 2A and D). Consistent with this trend, the specific heme content under glucose conditions was 0.13 ± 0.01 mg/g DCW, whereas under xylose conditions it significantly increased to 0.25 ± 0.02 mg/g DCW (*p* = 0.0027; Fig. S1 in Additional file 1). Likewise, heme titers were 1.9 ± 0.2 mg/L in glucose cultures and 4.0 ± 0.3 mg/L in xylose cultures, representing a significant increase under xylose conditions (*p* = 0.0005; Fig. S2). Overall, both specific heme content and volumetric heme titer were approximately two-fold higher under xylose than under glucose conditions. During xylose utilization, reduced ethanol overflow and increased respiratory metabolism likely promoted higher biomass formation and elevated intracellular metabolite pools (Lee et al. 2021; Barros et al. 2024).

Heme biosynthesis is tightly coupled to respiratory activity because multiple components of mitochondrial electron transport require heme as a prosthetic group. Under glucose conditions, Crabtree-positive *S. cerevisiae* represses respiration and favors fermentative metabolism, which limits mitochondrial biogenesis and the cellular demand for heme. In contrast, xylose metabolism in engineered strains partially alleviates glucose repression, promoting respiratory flux, mitochondrial function, and the tricarboxylic acid (TCA) cycle (Jin et al. 2004; Yun et al. 2018; Lee et al. 2021). This metabolic state can enhance the supply of heme precursors, such as succinyl-CoA and glycine, and increase the demand for heme-containing respiratory complexes, thereby stimulating heme biosynthesis. The higher intracellular heme levels observed during xylose fermentation are therefore consistent with a respiration-associated metabolism that supports both increased biomass formation and elevated heme accumulation.

### Effect of glucose/xylose co-utilization and fed-batch feeding on heme production in shake-flask fermentation

**Fig. 3:**
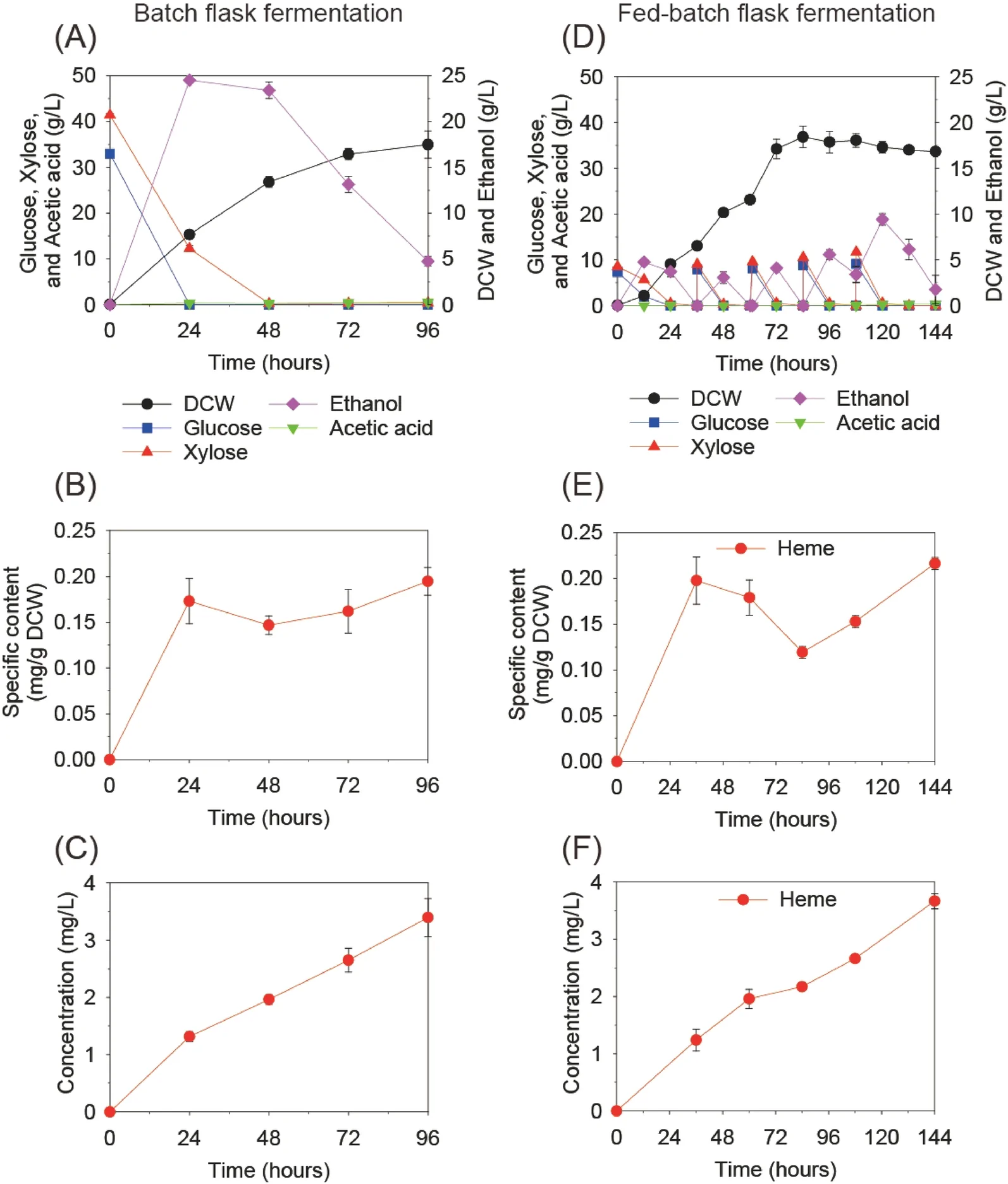
Mixed-sugar shake-flask fermentation: comparison of batch and fed-batch operation for biomass formation and heme production. *S. cerevisiae* SR8 was cultivated in shake flasks using a mixed-sugar substrate (40 g/L glucose + 40 g/L xylose) under batch (**A**–**C**) or fed-batch (**D**–**F**) conditions. Time-course profiles of biomass accumulation (DCW), sugar consumption (glucose and xylose), and byproduct formation (ethanol and acetic acid) during batch (**A**) and fed-batch (**D**) fermentation. DCW and ethanol are plotted on the right y-axis, and sugars and acetic acid are plotted on the left y-axis. Specific heme content over time during batch (**B**) and fed-batch (**E**) fermentation. Volumetric heme concentration during batch (**C**) and fed-batch (**F**) fermentation. Experiments were performed in triplicate, and error bars represent standard deviations from each cultivation

*Miscanthus × giganteus* hydrolysates contain mixed carbon sources, primarily glucose and xylose, along with acetic acid. To evaluate heme production on co-utilization of glucose and xylose relative to single-sugar conditions, *S. cerevisiae* SR8 was cultivated in culture media containing 40 g/L glucose and 40 g/L xylose. Growth on mixed sugars improved biomass and volumetric heme production compared to glucose, but resulted in lower specific heme content than xylose (Figs. 2A, D and 3A). Under the mixed-sugar condition, the specific heme content reached 0.19 ± 0.02 mg/g DCW, which was higher than that obtained with glucose alone (0.13 ± 0.01 mg/g DCW) but lower than that obtained with xylose alone (0.25 ± 0.02 mg/g DCW) (Figs. 2B, E and 3B). Similarly, the volumetric heme titer under mixed sugars was 3.40 ± 0.09 mg/L, higher than that in glucose-only cultures (1.9 ± 0.2 mg/L) and slightly lower than that in xylose-only cultures (4.0 ± 0.3 mg/L) (Figs. 2C, F and 3C). The presence of glucose under mixed-sugar conditions led to fermentative metabolism, as indicated by increased ethanol accumulation compared to the xylose condition. Therefore, the presence of glucose diminishes the advantage of xylose metabolism in avoiding the Crabtree effect (Jin et al. 2012; Shin et al. 2024).

To determine whether controlling glucose availability could decrease ethanol accumulation and fermentative metabolism, shake-flask fed-batch fermentation was conducted by supplying the mixed-sugar solution in small aliquots. At the end of fed-batch fermentation, the specific heme content increased from 0.19 ± 0.02 to 0.22 ± 0.01 mg/g DCW, although the difference was not statistically significant (*p* = 0.087; Figs. 3B, E and S1). Likewise, the increase in volumetric heme titer was not significant (from 3.40 ± 0.09 mg/L in batch to 3.7 ± 0.1 mg/L in fed-batch; *p* = 0.349; Figs. 3C, D, F and S2). These results indicate that the benefit of fed-batch feeding could not be clearly demonstrated in shake-flask cultivation, likely due to limited oxygen transfer. Therefore, bioreactor fermentations were performed to assess the effect of fed-batch feeding under controlled aeration.

In principle, fed-batch operation is expected to reduce metabolic inhibition by preventing rapid accumulation of ethanol and other byproducts, thereby supporting higher biomass formation and improved product yields. However, shake-flask cultivation is inherently limited in oxygen transfer, particularly at high cell density. As a result, the potential benefits of fed-batch feeding could not be fully evaluated in shake flask cultivations.

### Comparison of batch and fed-batch bioreactor fermentations using a mock hydrolysate sugar mixture

A mock hydrolysate was prepared to match the sugar and inhibitor composition of our *Miscanthus × giganteus* hydrolysates, containing 76 g/L glucose, 64 g/L xylose, and 7 g/L acetic acid in the yeast-defined medium described in a previous study (van Hoek et al. 2000). Fermentations were conducted in a 2 L bioreactor using a total working volume of 500 mL. For batch fermentation, the full 500 mL of the mock hydrolysate was added at the start time. For fed-batch fermentation, 100 mL of the mock hydrolysate was added at 0 h, 6 h, 12 h, 18 h, and 24 h. Agitation speed was maintained at 500 rpm, and aeration was supplied at 1 VVM.

**Fig. 4:**
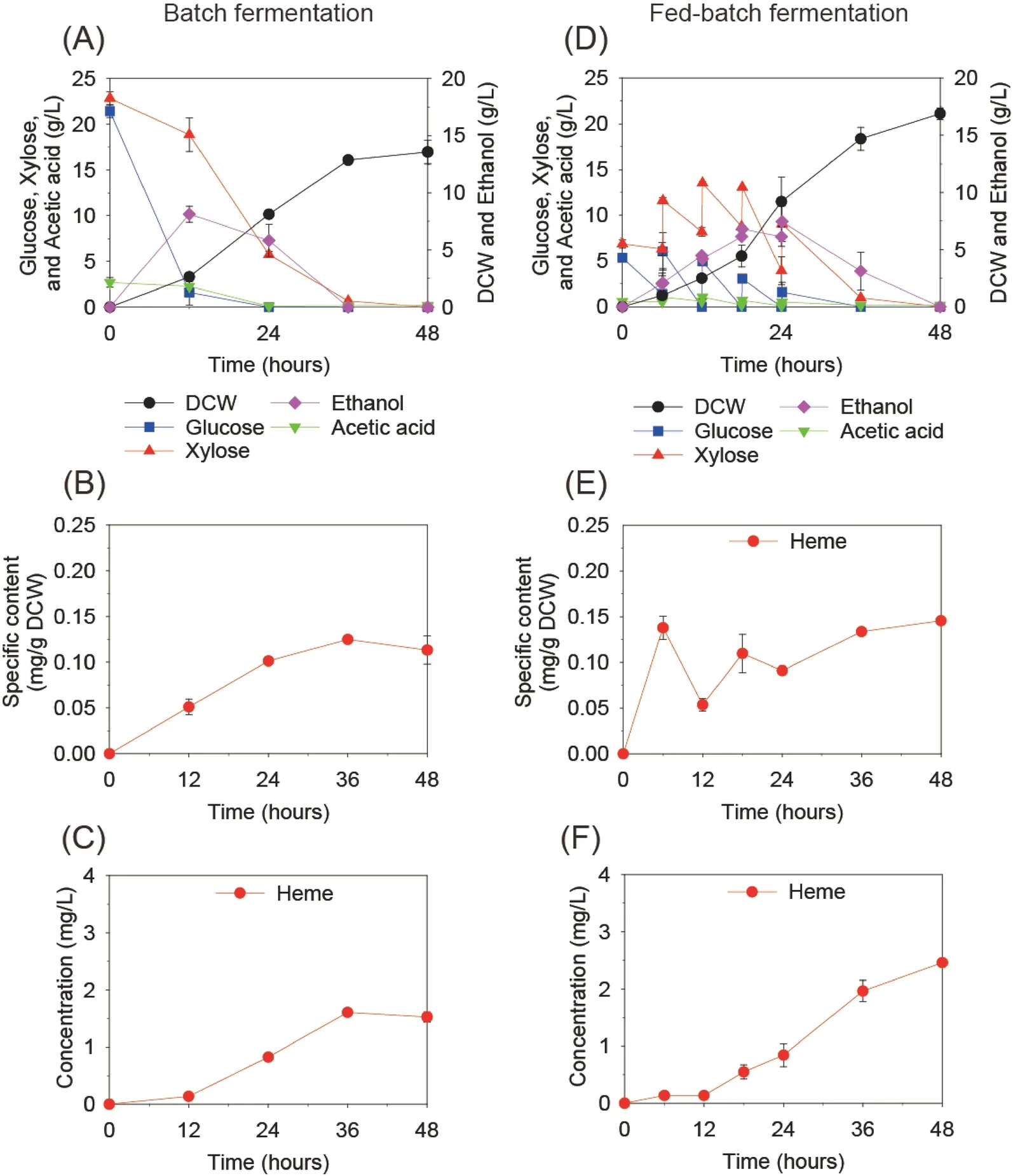
Bioreactor batch and fed-batch fermentations using a mock hydrolysate sugar mixture. Batch (**A**–**C**) and fed-batch (**D**–**F**) fermentations of *S. cerevisiae* SR8 were performed in a 2-L bioreactor using a mock hydrolysate formulation containing glucose, xylose, and acetic acid. Time-course profiles of *S. cerevisiae* SR8 during batch (**A**) and fed-batch (**D**) fermentation. Specific heme content during batch (**B**) and fed-batch (**E**) fermentation. Volumetric heme concentration during batch (**C**) and fed-batch (**F**) fermentation. Experiments were performed in triplicate, and error bars represent standard deviations from each cultivation

Biomass formation was significantly higher under fed-batch fermentation than under batch fermentation, reaching 16.9 ± 0.5 and 13.60 ± 0.04 g/L DCW, respectively (*p* = 0.0064; Fig. S1) (Fig. 4A and D). Specific heme content significantly increased from 0.11 ± 0.02 mg/g DCW in batch culture to 0.150 ± 0.004 mg/g DCW in fed-batch culture (*p* = 0.022; Fig. S1) (Fig. 4B and E). Similarly, volumetric heme concentration significantly rose from 1.53 ± 0.09 mg/L to 2.50 ± 0.01 mg/L under fed-batch fermentation (*p* = 5.04 × 10^− 5^; Fig. S2) (Fig. 4C and F). Overall, fed-batch operation increased DCW, specific heme content, and volumetric heme titer by 24%, 36%, and 63%, respectively, relative to batch fermentation, demonstrating that fed-batch bioreactor cultivation improved heme production from the mock hydrolysate sugar mixture.

These results indicate that incremental sugar addition, which lowered the effective glucose concentration in the broth, enhanced biomass accumulation and improved heme production. Collectively, fed-batch fermentation provides an effective strategy for heme production on mixed-sugar substrates, such as lignocellulosic hydrolysates.

### Demonstration of fed-batch bioreactor fermentation using lignocellulosic hydrolysates

**Fig. 5:**
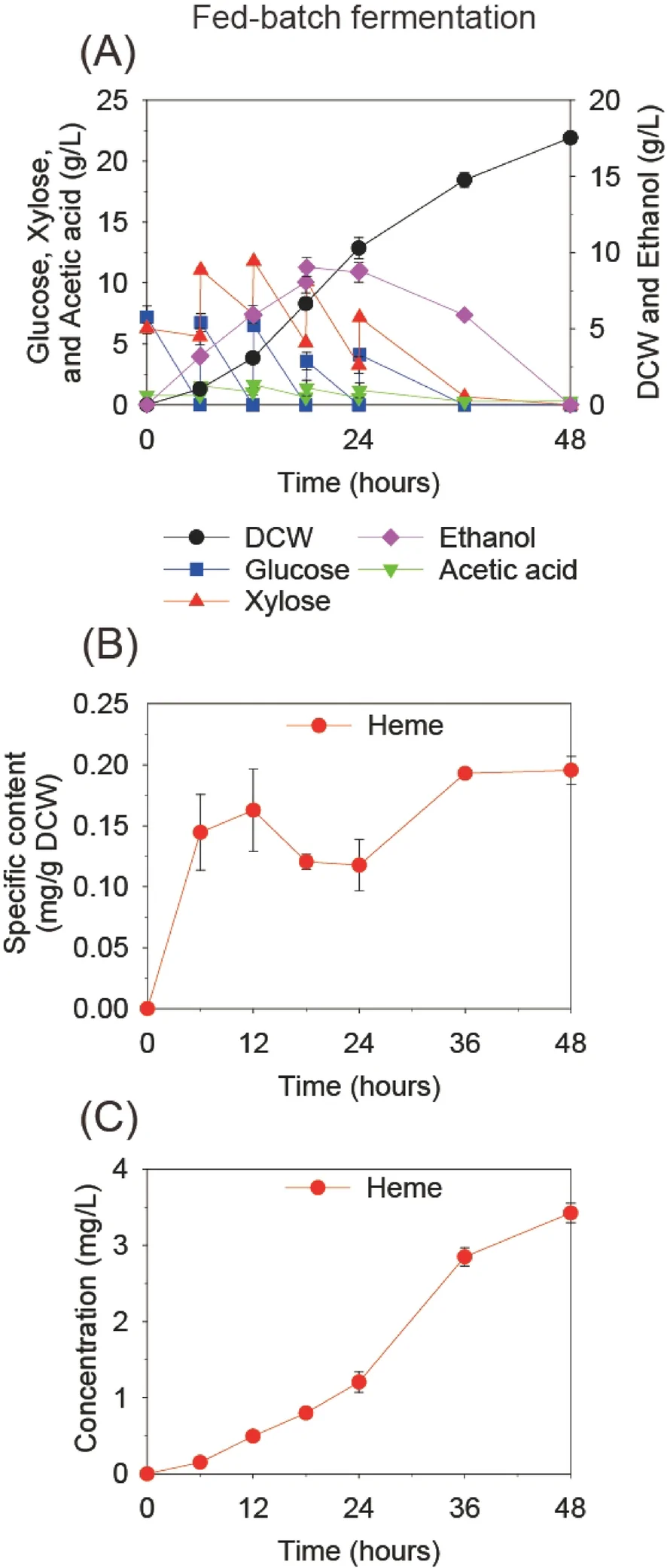
Fed-batch bioreactor fermentation using *Miscanthus* × *giganteus* lignocellulosic hydrolysate. Fed-batch cultivation of *S. cerevisiae* SR8 was performed in a 2-L bioreactor using *Miscanthus × giganteus* lignocellulosic hydrolysate containing glucose, xylose, and acetic acid. Time-course profiles of *S. cerevisiae* SR8 during fermentation (**A**). Specific heme content during fermentation (**B**). Volumetric heme concentration during fermentation (**C**). Experiments were performed in triplicate, and error bars represent standard deviations from each cultivation

To evaluate fermentation directly on lignocellulosic hydrolysates, we performed fed-batch cultivation with *Miscanthus × giganteus* hydrolysates as the substrate, applying the same feeding strategy used for the mock hydrolysate. Biomass accumulated to 17.5 ± 0.4 g/L DCW, representing a 4% increase compared with the mock hydrolysate condition, although this difference was not statistically significant (*p* = 0.0706) (Fig. 5A). The specific heme content reached 0.20 ± 0.01 mg/g DCW, corresponding to a 33% increase relative to the mock hydrolysate fed-batch condition (0.150 ± 0.004 mg/g DCW, *p* = 0.0007; Fig. S1) (Fig. 5B). Similarly, the volumetric heme titer reached 3.4 ± 0.1 mg/L, corresponding to a 37% increase relative to the mock hydrolysate fed-batch condition (2.50 ± 0.01 mg/L, *p* = 4.88 × 10^− 5^; Fig. S2) (Fig. 5C). Overall, compared with fed-batch fermentation using the mock hydrolysate, fed-batch fermentation with *Miscanthus × giganteus* hydrolysates numerically increased DCW, specific heme content, and volumetric heme titer, with significant improvements in both specific heme content and volumetric heme production. This improvement may be attributable to additional nutrients or growth-promoting components present in the hydrolysates. This nutritional benefit of lignocellulosic hydrolysates has rarely been examined, as most fermentation studies with these substrates have focused on producing target chemicals rather than yeast biomass.

### Functional evaluation of hydrolysate-derived yeast extract as a microbial growth supplement

**Fig. 6:**
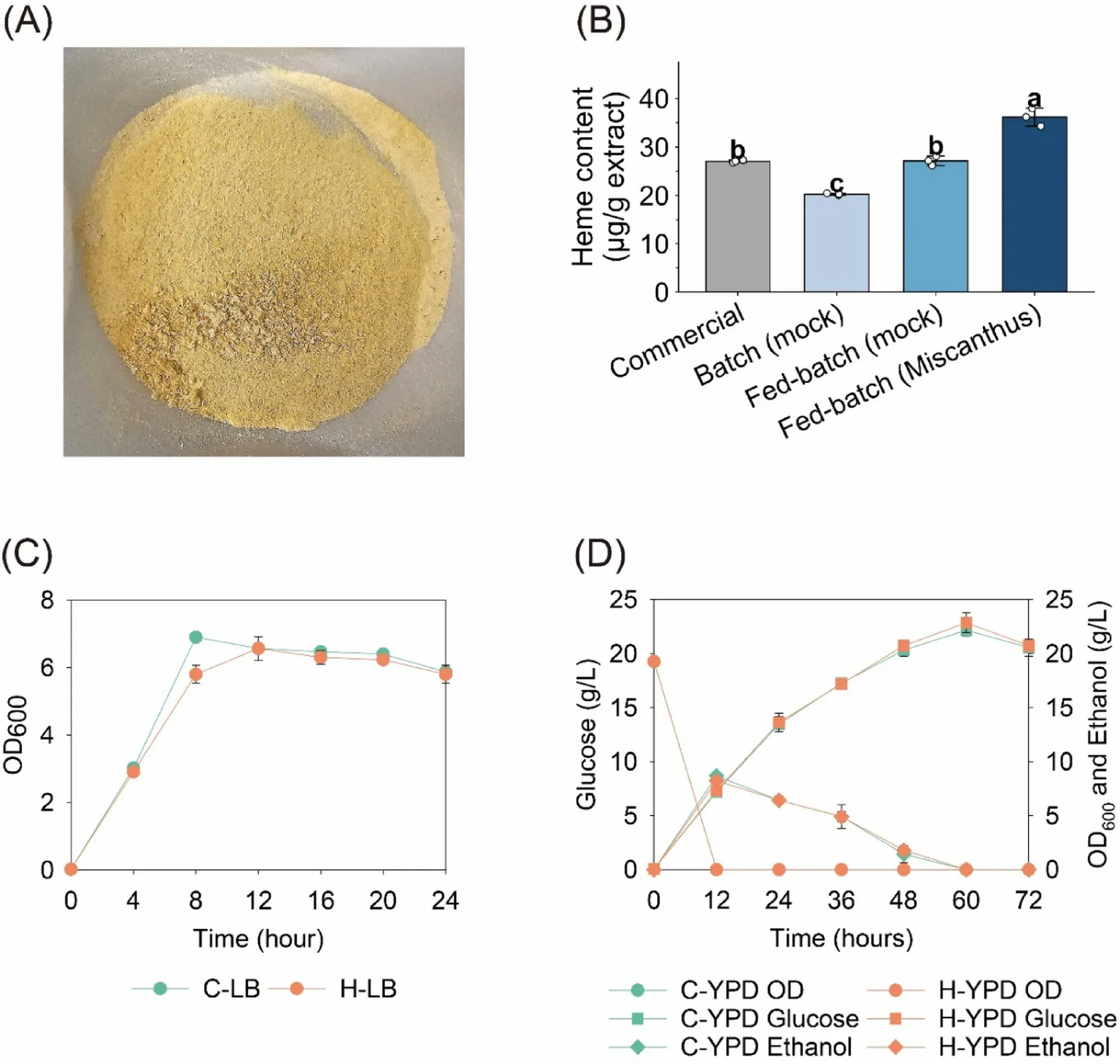
Production and evaluation of hydrolysate-derived, heme-enriched yeast extract. Representative image of the lyophilized yeast extract produced from *S. cerevisiae* SR8 biomass via autolysis (**A**). Specific heme content (µg/g yeast extract) of yeast extracts prepared from batch mock hydrolysate fermentation, fed-batch mock hydrolysate fermentation, and fed-batch *Miscanthus × giganteus* lignocellulosic hydrolysate fermentation, compared with a commercial yeast extract. Bars represent mean ± standard deviation of three biological replicates, with individual replicate values shown as open circles. Different lowercase letters above bars indicate significantly different groups (one-way ANOVA followed by Tukey’s honestly significant difference (HSD) post-hoc test, *p* < 0.05; “a” denotes the group with the highest mean) (**B**). Growth profile of *E. coli* in LB prepared with commercial yeast extract (C-LB) or hydrolysate-derived yeast extract (H-LB) (**C**). Growth profile of *S. cerevisiae* in YPD prepared with commercial yeast extract (C-YPD) or hydrolysate-derived yeast extract (H-YPD) (**D**). This test was performed in triplicate, and error bars indicate standard deviations from each cultivation

The functionality of hydrolysate-derived yeast extract was evaluated by comparing its ability to support microbial growth with that of commercial yeast extract. H-YE supported the growth of both *E. coli* and *S. cerevisiae* at levels comparable to those observed with C-YE (Fig. 6C, D).

To further contextualize this growth-supporting functionality, the compositional profiles of H-YE and C-YE were compared (Table 1). C-YE contained higher levels of soluble protein than H-YE (235 ± 15 vs. 183 ± 9 mg/g; p-value = 0.006), and its total carbohydrate content was approximately three-fold higher than that of H-YE (30 ± 3 vs. 11 ± 2 mg glucose equivalents/g; p-value = 0.001). Total nucleic acid content was also higher in C-YE than in H-YE (175 ± 7 vs. 146 ± 3 mg/g; *p* = 0.002). In contrast, amino nitrogen content was not significantly different between C-YE and H-YE (196 ± 9 vs. 175 ± 11 mg glycine equivalents/g; *p* = 0.067).

**Table 1:** Composition of commercial and hydrolysate-derived yeast extracts

	Soluble protein (mg/g extract)	Amino nitrogen (mg glycine eq/g extract)	Total carbohydrate (mg glucose eq/g extract)	Total nucleic acid (mg/g extract)
C-YE	235 ± 15	196 ± 9	30 ± 3	175 ± 7
H-YE	183 ± 9	175 ± 11	11 ± 2	146 ± 3
*p*-value	0.006	0.067	0.001	0.002
Significance	**	ns	**	**

Soluble protein, amino nitrogen, total carbohydrate, and total nucleic acid contents are reported as mean ± standard deviation from three biologicalreplicates. Statistical differences between C-YE and H-YE were determined by two-tailed Student’s t-test. “ns” indicates no statistically significantdifference; *, p < 0.05; **, p < 0.01; ***, p < 0.001

Although H-YE contained lower absolute levels of several macromolecular components, the preservation of amino nitrogen content is consistent with the comparable growth performance. These results suggest that the single-step autolysis process generated a yeast extract with growth-supporting functionality comparable to that of commercial yeast extract under the tested cultivation conditions.

### Heme enrichment and downstream recovery during yeast extract preparation

Yeast extract derived from fed-batch cultivation on lignocellulosic hydrolysates contained significantly higher heme content than C-YE (Tukey HSD, *p* < 0.05). The heme content of yeast extracts prepared from batch mock hydrolysate, fed-batch mock hydrolysate, fed-batch lignocellulosic hydrolysates, and commercial yeast extract was 20.3 ± 0.2, 27 ± 1, 36 ± 2, and 27.1 ± 0.3 µg/g yeast extract, respectively (Fig. 6B). The heme content of the yeast extracts followed the same general trend observed for the specific heme content of the corresponding yeast biomass. These results demonstrate that *Miscanthus* hydrolysate-derived yeast biomass can be converted into a yeast extract with enhanced heme content relative to the commercial yeast extract tested in this study.

To place these values in an application-relevant context, Maini Rekdal et al. reported that Impossible™ Burger contains approximately 150 µg heme/g dry weight based on LC–MS analysis (Maini Rekdal et al. 2024). Using the moisture content reported for Impossible Burger Patties in USDA FoodData Central (FDC ID 1997200) (U.S. Department of Agriculture, Agricultural Research Service 2021), corresponding to approximately 62.8% moisture and 37.2% dry matter, this value corresponds to approximately 56 µg heme/g wet product, or 5.6 mg heme per 100 g patty. In comparison, the highest heme content obtained in the present study was 36 ± 2 µg/g yeast extract. At a hypothetical inclusion level of 2% (w/w) in a food formulation, this yeast extract would contribute approximately 72 µg heme per 100 g product. Therefore, matching the estimated heme level of Impossible™ Burger would require a yeast extract heme content of approximately 2.8 mg/g, corresponding to a ~ 80-fold increase relative to the current material. Thus, while the present H-YE should not yet be viewed as a stand-alone heme color or flavor ingredient at commercial inclusion levels, it demonstrates the feasibility of producing a nutritionally functional, heme-enriched yeast extract from lignocellulosic hydrolysates. Further improvements in intracellular heme accumulation, extract recovery, and downstream concentration would be required to develop this platform toward food-ingredient applications.

The application-level gap described above prompted us to examine whether downstream processing, rather than intracellular heme accumulation alone, may represent a major bottleneck for producing heme-enriched yeast extract. In the present process, the yeast extract yield from *S. cerevisiae* biomass produced on lignocellulosic hydrolysates was approximately 20%, corresponding to 0.2 g yeast extract per g DCW. Because the dried cells contained 0.2 mg heme/g DCW, complete retention of cellular heme during yeast extract production would theoretically yield approximately 1 mg heme/g yeast extract. However, the measured heme content in the final autolysis-derived yeast extract was only 0.036 mg/g, representing an approximately 28-fold lower concentration than the theoretical maximum. This discrepancy indicates substantial heme loss or dilution during yeast extract production and identifies downstream recovery as a major bottleneck in producing heme-enriched yeast extract.

To identify a more effective extraction strategy for heme-enriched yeast extract production under standardized biomass conditions, we compared autolysis, autoclave-assisted extraction, and high-pressure homogenization (HPH) using YPD-grown yeast biomass as a model substrate (Table 2). Heme partitioning varied markedly among the three methods. In the autolysis process, 49.6 ± 0.3% of the initial cellular heme remained associated with the insoluble debris fraction, whereas only 1.55 ± 0.01% was recovered in the soluble extract fraction. Autoclave-assisted extraction decreased the debris-associated heme fraction to 16.3 ± 0.8%; however, the unrecovered heme fraction increased to 81 ± 1%, suggesting substantial heme loss during thermal processing. In contrast, HPH further reduced debris-associated heme to 12 ± 2% and significantly increased soluble extract-associated heme to 9.4 ± 0.2%. Consequently, HPH achieved the highest final extract heme recovery, reaching 3.70 ± 0.04%, compared with 0.480 ± 0.001% for autolysis and 0.51 ± 0.09% for autoclave-assisted extraction. These results indicate that mechanical disruption by HPH is more effective than autolysis or autoclave-assisted extraction for releasing cellular heme into the soluble yeast extract fraction.

**Table 2:** Heme partitioning after different cell disruption methods

Extraction method	Heme retained in insoluble debris (%)	Heme recovered in soluble extract fraction (%)	Unrecovered heme (%)	Final extract heme recovery (%)
Autolysis	49.6 ± 0.3^a^	1.55 ± 0.01^c^	48.9 ± 0.3^b^	0.480 ± 0.001^b^
Autoclave	16.3 ± 0.8^b^	2.9 ± 0.3^b^	81 ± 1^a^	0.51 ± 0.09^b^
HPH	12 ± 2^c^	9.4 ± 0.2^a^	79 ± 3^a^	3.70 ± 0.04^a^

Values are presented as mean ± standard deviation from three biological replicates. Heme distribution was calculated relative to the total hemecontent in the initial cell biomass, which was set as 100%. Different superscript lowercase letters within the same column indicate statistically significantdifferences among extraction methods, as determined by one-way ANOVA followed by Tukey’s HSD test (p < 0.05)

Although the overall heme recovery remained low even with HPH, the higher recovery achieved by this method identifies cell disruption and downstream clarification as critical targets for further optimization. Therefore, future development of heme-enriched yeast extract should employ HPH-based processing or related mechanical disruption strategies to improve heme release and retention, while also increasing intracellular heme accumulation through strain engineering approaches such as enhancement of heme biosynthetic flux, iron uptake, and mitochondrial heme assembly. The resulting yeast extract should therefore be evaluated in relevant food matrices to determine whether it provides measurable color, color stability, flavor-related, or formulation effects. Together, these findings establish both a lignocellulose-based fermentation route for producing heme-enriched yeast biomass and a downstream processing direction for improving heme recovery in yeast extract production.

## Conclusion

This study demonstrates a scalable strategy for producing heme-enriched *Saccharomyces cerevisiae* biomass and yeast extract from lignocellulosic hydrolysates derived from *Miscanthus × giganteus*. Xylose improved biomass formation and intracellular heme accumulation relative to glucose. However, this advantage was diminished in the presence of glucose due to the Crabtree effect. Therefore, fed-batch bioreactor cultivation further improved heme production under controlled glucose concentration and aeration conditions. Fermentation using *Miscanthus × giganteus* hydrolysates outperformed a mock hydrolysate formulation, highlighting the potential benefits of lignocellulosic substrates for yeast-based bioprocesses. Yeast extract derived from hydrolysate-grown biomass supported microbial growth comparably to commercial yeast extract under the tested conditions.

Comparison of extraction methods further showed that HPH improved heme release into the soluble yeast extract fraction and achieved the highest final extract heme recovery, identifying mechanical disruption as a key downstream optimization target. Although additional improvements in intracellular heme accumulation and downstream processing will be required to meet application-level targets, this work establishes a proof-of-concept platform for producing heme-enriched yeast ingredients directly from renewable lignocellulosic biomass. More broadly, the results illustrate an alternative lignocellulose valorization pathway beyond traditional biofuel and bulk chemical production, expanding the potential of lignocellulosic feedstocks for the sustainable manufacturing of functional food ingredients. Because the hydrolysate was derived from non-food lignocellulosic biomass, further compositional and safety analyses, including heavy metal, pesticide residue, and contaminant screening, will be required before food applications.

## Supplementary Information

Below is the link to the electronic supplementary material.

Supplementary Material 1

## Data Availability

The dataset(s) supporting the conclusions of this article are included within the article and its additional file(s). Additional raw data are available from the corresponding author upon reasonable request.

## Abbreviations

C-YE: Commercial yeast extract
DCW: Dry cell weight
DDW: Double-distilled water
HPH: High-pressure homogenization
H-YE: Hydrolysate-derived yeast extract
OD: Optical density
PPIX: Protoporphyrin IX
VVM: Volume of air per volume of liquid per minute
