# Supplementary material for: Valorization of Miscanthus × giganteus hydrolysates into heme-enriched yeast biomass and extract using xylose-fermenting Saccharomyces cerevisiae
Source: Bioresour Bioprocess. 2026 Sep 15;13(1):137. doi: 10.1186/s40643-026-01133-1 (PMC13578150; doi:10.1186/s40643-026-01133-1)
Supplement: Supplementary file 1 [file 40643_2026_1133_MOESM1_ESM.docx]

*Supplementary Information*

**Valorization of Lignocellulosic Hydrolysates into Heme-Enriched Yeast Extract Using Xylose-Fermenting *Saccharomyces cerevisiae***

Suk-Chae Jung^1,†^, Hyunjoon Oh^2,3,†^, Sungjun Kim^2,3^, Shuchi Singh^3,4^, Vijay Singh^3,4^, Soo Rin Kim^5^, Ting Lu^6,7^, Hyun Gi Koh^8,*^, and Yong-Su Jin^2,3,6,9,*^

^1^Department of Chemical and Biomolecular Engineering, University of Illinois, Urbana-Champaign, Urbana, IL, 61801, USA

^2^Department of Food Science and Human Nutrition, University of Illinois, Urbana-Champaign, Urbana, IL, 61801, USA

^3^DOE Center for Advanced Bioenergy and Bioproducts Innovation, University of Illinois, Urbana-Champaign, Urbana, 61801, IL, USA

^4^Department of Agricultural and Biological Engineering, University of Illinois, Urbana-Champaign, Urbana, IL, USA

^5^School of Food Science and Biotechnology, Kyungpook National University, Daegu 41566, Korea

^6^Carl R. Woese Institute for Genomic Biology, University of Illinois, Urbana-Champaign, Urbana, IL, 61801, USA

^7^Department of Bioengineering, University of Illinois, Urbana-Champaign, Urbana, IL, 61801, USA

^8^Department of Biological and Chemical Engineering, Hongik University, Sejong 30016, Republic of Korea

^9^Centre for Precision Fermentation and Sustainability (PreFerS), Illinois Advanced Research Center at Singapore (Illinois ARCS), Singapore

^†^These authors contributed to this work equally.

***Corresponding author:**

Yong-Su Jin ([ysjin@illinois.edu](mailto:ysjin@illinois.edu)); Hyun Gi Koh (hgkoh@hongik.ac.kr)

**Figure S1. Specific heme content (mg/g DCW) across four pairwise comparisons of fermentation conditions in *Saccharomyces cerevisiae* SR8.** (Panel 1) Glucose vs. xylose as sole carbon source in shake-flask cultivation. (Panel 2) Batch vs. fed-batch operation in mixed-sugar (glucose + xylose) shake-flask cultivation. (Panel 3) Batch vs. fed-batch operation in 2-L bioreactor cultivation on mock hydrolysate. (Panel 4) Fed-batch bioreactor cultivation on mock hydrolysate vs. *Miscanthus × giganteus* hydrolysate. Bars represent mean ± standard deviation (n = 3 biological replicates), with individual replicate values shown as open circles. Statistical significance was assessed by two-tailed Student's *t*-test: *, *p*-value < 0.05; **, *p*-value < 0.01; ***, *p*-value < 0.001; ns, not significant.

**Figure S2. Volumetric heme titer (mg/L) across four pairwise comparisons of fermentation conditions in *S. cerevisiae* SR8.** (Panel 1) Glucose vs. xylose as sole carbon source in shake-flask cultivation. (Panel 2) Batch vs. fed-batch operation in mixed-sugar (glucose + xylose) shake-flask cultivation. (Panel 3) Batch vs. fed-batch operation in 2-L bioreactor cultivation on mock hydrolysate. (Panel 4) Fed-batch bioreactor cultivation on mock hydrolysate vs. *Miscanthus × giganteus* hydrolysate. Bars represent mean ± standard deviation (n = 3 biological replicates), with individual replicate values shown as open circles. Statistical significance was assessed by two-tailed Student's *t*-test: *, *p*-value < 0.05; **, *p*-value < 0.01; ***, *p*-value < 0.001; ns, not significant.
